# Risk of cancer in the vicinity of municipal solid waste incinerators: importance of using a flexible modelling strategy

**DOI:** 10.1186/1476-072X-8-31

**Published:** 2009-05-28

**Authors:** Sarah Goria, Côme Daniau, Perrine de Crouy-Chanel, Pascal Empereur-Bissonnet, Pascal Fabre, Marc Colonna, Cedric Duboudin, Jean-François Viel, Sylvia Richardson

**Affiliations:** 1 Institute of Public Health Surveillance (InVS), Saint-Maurice, France; 2 Isère Cancer Registry, Myelan, France; 3 French Agency for Environmental and Occupational Health Safety (Afsset), Maisons-Alfort, France; 4 CNRS n 6249 "Chrono-Environment", Faculty of Medicine, Besançon, France; 5 Department of Epidemiology and Public Health, Imperial College London, London, UK

## Abstract

**Background:**

We conducted an ecological study in four French administrative departments and highlighted an excess risk in cancer morbidity for residents around municipal solid waste incinerators. The aim of this paper is to show how important are advanced tools and statistical techniques to better assess weak associations between the risk of cancer and past environmental exposures.

**Methods:**

The steps to evaluate the association between the risk of cancer and the exposure to incinerators, from the assessment of exposure to the definition of the confounding variables and the statistical analysis carried out are detailed and discussed. Dispersion modelling was used to assess exposure to sixteen incinerators. A geographical information system was developed to define an index of exposure at the IRIS level that is the geographical unit we considered.

Population density, rural/urban status, socio-economic deprivation, exposure to air pollution from traffic and from other industries were considered as potential confounding factors and defined at the IRIS level. Generalized additive models and Bayesian hierarchical models were used to estimate the association between the risk of cancer and the index of exposure to incinerators accounting for the confounding factors.

**Results:**

Modelling to assess the exposure to municipal solid waste incinerators allowed accounting for factors known to influence the exposure (meteorological data, point source characteristics, topography). The statistical models defined allowed modelling extra-Poisson variability and also non-linear relationships between the risk of cancer and the exposure to incinerators and the confounders.

**Conclusion:**

In most epidemiological studies distance is still used as a proxy for exposure. This can lead to significant exposure misclassification. Additionally, in geographical correlation studies the non-linear relationships are usually not accounted for in the statistical analysis. In studies of weak associations it is important to use advanced methods to better assess dose-response relationships with disease risk.

## Background

**Figure 1:**
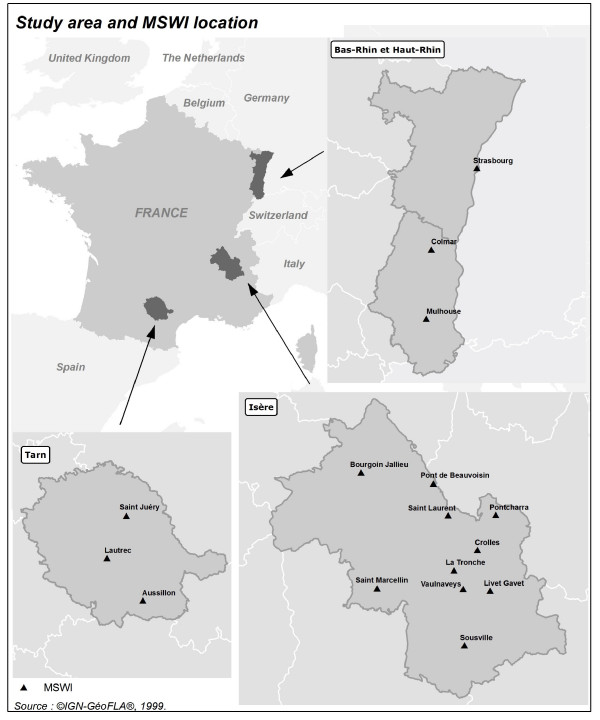
**The 4 French departments included in the study and their 16 municipal solid waste incinerators**. There were 10 MSWIs in Isère, 1 in Bas Rhin, 2 in Haut Rhin and 3 in Tarn.

We highlighted an excess risk in cancer morbidity for residents around municipal solid waste incinerators (MSWIs). The study took place in 4 French departments (administrative subdivisions of a region in France), Isère, Bas Rhin, Haut Rhin and Tarn (Figure 1), provided with a general cancer registry. All cancers and 6 selected subtypes (breast, lung, liver, non-Hodgkin's lymphoma, soft-tissue sarcoma and leukaemia) registered between 1990 and 1999 on adults over 14 years of age were considered. During this study period, around 135,000 cases of primary cancer were identified among approximately 25,000,000 person-years. The study was based on 2270 small geographical units, the IRIS, that are used in population census. The home address at the moment of diagnosis was used to locate each case in an IRIS. Thus we supposed that cases lived in the same IRIS during the exposure period.

Latency periods of 10 and 5 years were used between the exposure period and cancer incidence, respectively for solid tumors and leukaemias. The oldest incinerator started in 1972. Thus, the exposure periods were defined as going from 1972 to 1985 and from 1972 to 1990, respectively for solid tumors and leukaemias.

Exposure to incinerators that operated at least one year from 1972 to 1990 was estimated from a second generation Gaussian dispersion model. These data were integrated in a geographical information system (GIS) to match location and estimated exposure.

Urbanisation (taken into account by: population density and rural/urban status), socio-economic deprivation, exposure to air pollution from road traffic and exposure to air pollution from other industries were considered as potential confounding factors. They were defined at the IRIS level.

To assess the association between the risk of cancer and past exposure to MSWIs, a Poisson regression analysis was performed taking into account the confounding factors. In particular, the study of the dose-response relationship was performed through the implementation of generalized additive models (GAM). Additionally, a Bayesian hierarchical analysis was used to account for overdispersion, spatially and non spatially structured. A department's effect was always included in the models to take into account the baseline risk of each department.

The aim of this paper is to describe the tools and methods used in this ecological study and to show how advanced GIS and statistical techniques were used to better assess dose-response relationships with disease risk. The paper by Fabre et al. (submitted) reviews the scientific literature on cancer and atmospheric emissions from incinerators, presents the associations found, compares them to those of previous studies and discusses this type of epidemiological study. The paper by Viel et al. [1] presents and discusses the results for non-Hodgkin's lymphoma.

## Methods

### The geographical unit: the IRIS

The geographical unit is the IRIS. An IRIS is the smallest unit for which different socio-economic and demographic data were available. It was defined by the National Institute for Statistics and Economic Studies (INSEE) to have demographic units of around 2,000 inhabitants. For this, INSEE partitioned all the urban *communes *(official municipalities, of any size) with more than 10,000 inhabitants and most of those of 5,000 to 10,000 inhabitants. For the other *communes *the IRIS coincides with the entire *commune*. The surface of the 2270 IRIS studied included in this study varies in the range 0.05 to 238 km^2 ^(the median is 3 km^2^). The 1995 over-14-years population varies in the range 2- 18380 inhabitants (the median is 660 inhabitants). The IRIS-level population for 1995 was estimated by diagonal interpolation [2] of the 1990 and 1999 national census counts as annual small area population counts were not available.

### Exposure to MSWIs

Sixteen MSWIs operated at least one year from 1972 to 1990. Figure 1 presents their distribution in the 4 departments included in this study. The incinerators in the neighbouring areas- close to the borders of the departments under study- were also considered, but did not contribute in the end because either their operating period or their exposure area did not overlap with ours.

We considered incinerators emissions of particles, metals and dioxins that is a mixture of dioxins-furanes and PCBs. No measurements were available of particles, metals and dioxins around the 16 incinerators for the period of interest. For this, the stack emissions of each incinerator were estimated retrospectively through expert assessments. The experts, representing operators, public authorities and a research institution, used a simplified version of the Delphi method [3], an iterative process towards consensus, and took into account the incinerators technical characteristics and their evolution over time (capacity, type of combustion, clearance and filtration processes). Given the technical processes of the 16 MSWIs included in this study, eight categories of incinerators were identified by the experts. An incinerator could be classified in several categories if there were important changes during its operation period. The estimations produced by the experts corresponded to a value of emission flow (*μ*g/Nm^3^) for each category. The flow values estimated for each of the 8 categories of incinerators were then multiplied by the annual tonnage of waste cremated by each incinerator: this gave the emission of every incinerator (*μ*g/s). These estimations were then used in the dispersion models.

**Figure 2:**
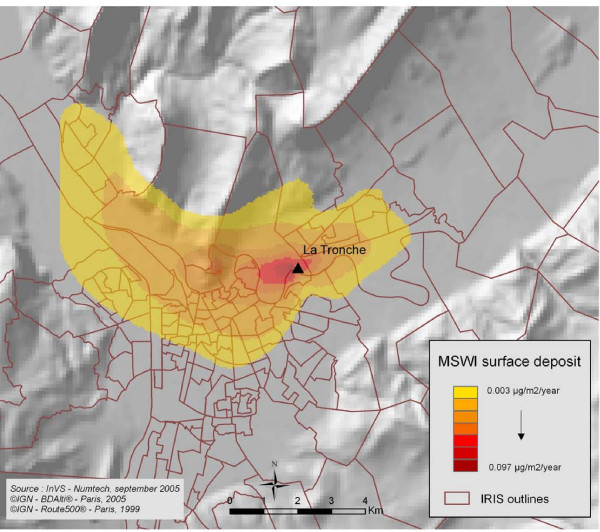
**Modelled surface deposit of dioxins (*μ*g/m^2^/year) around the incinerator of la Tronche-Isère department**.

A Gaussian model was used to model atmospheric dispersion and ground-level deposit within a square grid with unit cells of 200 m × 200 m, centred around the incinerators. The extent of the grid was adapted to the incinerators characteristics and environnement, ranging from 20 km × 20 km to 40 km × 40 km. This work was done with the software ADMS3 developed by CERC and UK Meteorological office . It is a second generation Gaussian model: it accounts for the changes in flow field and turbulence around complex terrain (hills) and uses them to compute concentrations. This was interesting as a few incinerators in the Isère department are located in valleys next to mountains as it can be seen in Figure 2. The parameters considered in the modelisation process are: estimations obtained from the experts, pollutant characteristics, stack height, meteorological data (wind speed and direction, temperature, atmospheric stability) and environmental characteristics such as surface topography and soil roughness.

Analysis focused on dioxins. At first three groups of carcinogenic pollutants emitted from MSWI stacks were studied (particles, metals and dioxins). As their emission flows and modelled atmospheric dispersion and deposition were highly correlated, we chose the dioxins as a proxy of pollutants emitted from MSWIs. The dioxins ground-level deposit (*μ*g/m^2^/year) served as a proxy of the annual dispersed release of pollutants from the incinerators.

Figure 2 shows the modelled ground-level deposit of dioxins around one of the incinerators included in the study. The interest of dispersion modelling versus concentric circles, that are often used to evaluate exposure to a point source of pollution, is clear: the exposure area is not symmetric around the incinerator. As shown by Hodgson et al. [4] using distance as a proxy for exposure can lead to significant exposure misclassification. Defining distance as a proxy for exposure takes no consideration of the point source characteristics, the meteorological conditions or the topographical features which play a significant role in determining dispersion and pollutant concentration. Dispersion modelling brings this supplementary information to the exposure measure.

#### GIS development

In order to estimate the population exposure to incinerators at the IRIS level a GIS was developed. It permitted to assign to each grid point of the ADMS3 modelisation an IRIS. As it can be seen in Figure 1, there are 10 incinerators in the Isère department and many of them are less than 20 km apart. Thus there are IRIS exposed to more than one MSWI. In this case the sum of the pollutant ground-level deposits for the points of the grids with the same coordinates was taken.

The value assigned to an IRIS was the median of all the points falling into the IRIS. The median number of points per IRIS is 19 (the number of points falling in an IRIS can go from 1 to a 1000). Of the 2270 IRIS, 520, that is 23%, lied in the modelisation grids. The IRIS that lie outside the grids were assigned the minimum value estimated in the modelisation grids.

This work was done with the ESRI ArcGIS 9.1.

#### Index of exposure

Dioxins persist in the environment and bioaccumulate. Thus the index of exposure was calculated to account for the number of years the plant had operated and the degradation speed in soils. It was defined as the mean of the cumulated ground-level deposits since the start of the plant activity (*μ*g/m^2^/year). It corresponds to the annual average of the deposits accumulated on the ground surface over all the duration of the incinerators' activity. It was obtained applying an exponential decreasing function with a half-life of 10 years for dioxins in the environment [5].

This ecological index of exposure does not take into account individual characteristics, such as residential history and time-activity patterns, leading to potential exposure misclassification.

The assessment of exposure to incinerators required an expert judgement to quantify stack emissions and then the modelling of atmospheric dispersion and surface deposition. This method is the best surrogate to measurements to assess retrospectively past emissions in a context of missing data. It is a real improvement for ecological studies. Indeed, the misclassification of exposure status is a methodological problem in this type of studies with small exposure effects.

### Confounding variables

We had no access to individual data on potential confounders. Five potential confounding factors available at the IRIS or *commune *level were taken into account: population density, rural/urban status, socio-economic level, exposure to road traffic pollution and to other polluting industries.

**Figure 3:**
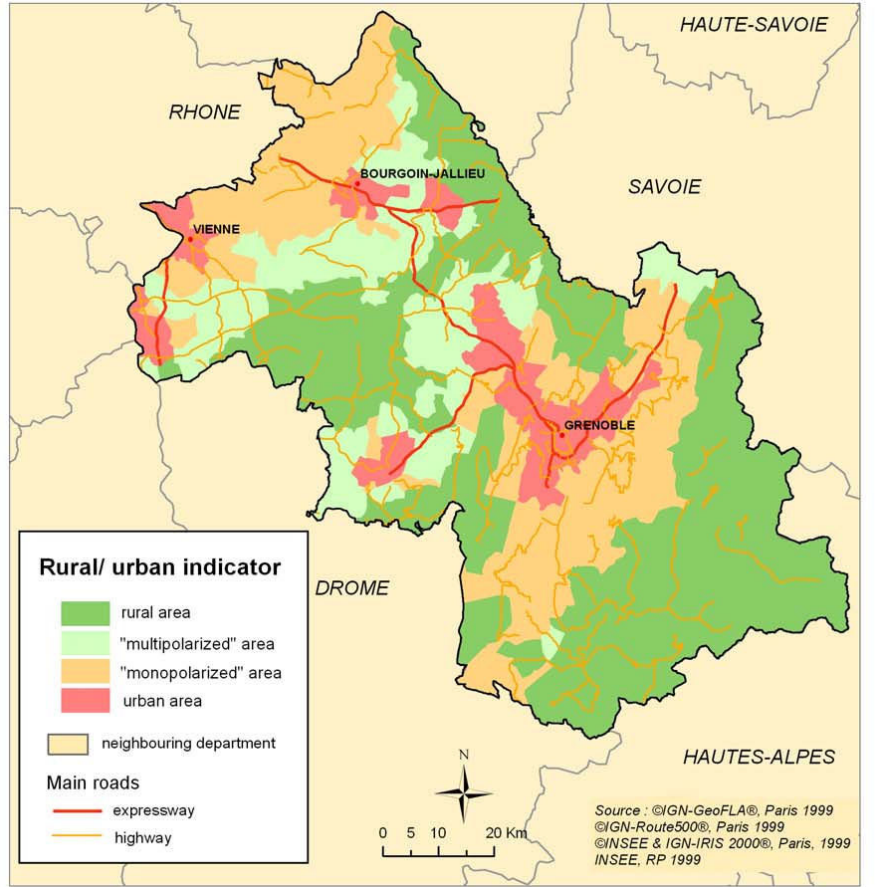
**Rural/urban indicator: Isère department**.

Using census information collected in 1990, the IRIS population density (inhabitants/km^2^) was computed. A rural/urban indicator was also available from INSEE (4 classes- from rural to urban- defined using data on population, home-work trajects and the area's job attractiveness). This variable was available at the *commune *level: the IRIS of a same *commune *were given the *commune*'s value of the rural/urban indicator. Figure 3 shows the rural/urban indicator for the Isère department.

Using census information of 1990, a socio-economic score was calculated for each IRIS by principal component analysis (PCA) of 6 socio-economic characteristics: the proportion of unemployed persons, the proportion of low social class (IV or V) households, the proportion of households without a car, the proportion of households who are not owner-occupied, the proportion of public households and the mean number of persons per room (for overcrowding). In the PCA the individual observations, that is the IRIS, were population weighted as is standard practice when analysing aggregated data [6]. The score was given by the first principal component. With the exception of the proportion of public households, the socio-economic indicators considered are those of the Townsend and Carstairs indexes [7,8]. These indicators were chosen so that their linear combination had a clear interpretation [6,9].

A proxy for exposure to road traffic pollution was defined by nitrogen dioxide (NO_2_) concentrations that were taken to be a marker of road traffic emitted cancerigenic pollutants. The data were obtained from the WHO study [10,11]: NO_2 _concentrations were estimated on a grid of 4 km × 4 km unit cells covering the whole territory of France. These estimations were obtained by cokriging using observed NO_2 _concentrations- year 2000- and information about land use. Using these data implies the strong hypothesis that the NO_2 _concentrations did not change between the '70s and '80s, that is the period of exposure, and the year 2000. We know that this is not the case locally, but we can suppose that globally the evolution of NO_2 _concentrations was relatively homogeneous. These data were implemented in the GIS to be used and to define the variable at the *commune *level: the IRIS of a same *commune *were given the *commune*'s value.

A proxy for exposure to other polluting industries was given by the number of industry-years per IRIS (for 1972–1985 or 1972–1990, data from INSEE). Only the industries emitting potential cancerigenic pollutants were taken into account.

The correlation coefficients (Spearman) between these variables range from 0.31 to 0.69. The highest coefficient is observed between population density and the rural/urban indicator. The correlation coefficients between each of these variables and the index of exposure to MSWIs range from 0.30 to 0.53. The highest coefficient is observed for the proxy of exposure to road traffic pollution.

In fine, the confounding variables considered are population density, the rural/urban indicator, the socio-economic score, the proxy of exposure to road traffic pollution and to other polluting industries. It is important to note that while the population data and the socio-economic variables were available at the IRIS level, the use of GIS was necessary to define the exposure to MSWIs and to traffic pollution.

Although we have accounted for several potential confounders, we cannot exclude the possibility of unmeasured confounding. The fact that the proxy for exposure to road traffic pollution was defined at the *commune *level and for the year 2000 and the fact that no individual information was available can lead to residual confounding.

### Statistical analysis

Since the observed number of cases are small, Poisson regressions models were fitted to assess the associations between the risk of cancer and the index of exposure to MSWIs. The models were fitted with an offset as the expected number of cancers. The expected number of cases for each IRIS were computed by applying reference incidence rates to the person-years of each IRIS stratified by age (5 year age classes) and gender. The reference rates were the incidence rates observed in the period 1990–1999 in 6 French departments-the 4 included in the study and 2 additional departments (Doubs and Hérault) also provided with a general cancer registry. The 2 additional departments were taken to have more stable reference rates.

#### Poisson regressions

Generalized additive models [12] were used. They are widely used in time-series studies of the health effects of air pollution [13]. These models are appropriate for exploring forms of associations between the risk of cancer and the exposure to MSWIs or the confounders without presupposing the shape, for example linear, *a priori*. This is of paramount importance as typically, there will be little prior indication for a specific parametric shape. To circumvent an over-simplistic linearity assumption, traditionally, strata of exposure are defined and a categorical analysis is performed. GAM regression models avoid the arbitrariness in the choice and the number of strata, combining full use of the quantitative aspect of the exposure measure with a flexible shape. To our knowledge, their use in geographical correlation studies is novel. We used GAMs with penalized cubic regression splines: the degree of smoothness of model terms is estimated as part of fitting [14,15].

The covariates were selected through the Akaike criterion [16].

The simplest approach to ecological analysis uses a multiple regression model for disease risk which only allows for Poisson variation. However, it is commonly observed that the variation not explained by the ecological variables (residual variation) might be substantially in excess of that expected from Poisson sampling theory. To acknowledge this, we proceeded in several steps.

At first, residual variation was taken into account by fitting a Poisson regression model allowing for overdispersion. After fitting standard Poisson regressions, we modelled the overdispersion in a hierarchical Bayesian framework which is well adapted to the analysis of disease risk on a small geographical scale [17-22]. It allows integrating, in the estimation of the unknown relative risks, local information consisting of the observed and expected number of cases in each area, the value of the variable of interest and of the potential confounding factors and prior information on the overall variability of the relative risks.

#### Hierarchical model

The approach we followed, suggested by Besag et al. [23], splits the extra-Poisson variation in two components. The first component of variation is spatially unstructured extra-Poisson variation, called heterogeneity. Modelling the heterogeneity variation allows for unmeasured variables that vary between areas in an unstructured way. The second component of variation, called clustering, varies smoothly across areas. Modelling the clustering variation allows for those unmeasured risk factors that vary smoothly with location.

The Deviance Information Criterion (DIC) [24] was used to compare models and decide whether the heterogeneity and clustering components had to be kept in the model or not. It was not always necessary to include both terms in the model. Models with smaller DIC are better supported by the data. The DIC for each model is the sum of the posterior mean deviance and a penalty related to an estimate of the "effective" number of parameters, *p*_*D*_. The *p*_*D *_term aims to capture the amount of shrinkage achieved by the hierarchical prior [25]. A value of *p*_*D *_that is small compared to the number of data points indicates that the prior provides a lot of information about the parameters and hence leads to considerable borrowing of strength across units.

As in the Besag et al. model, a Gaussian distribution was used as the prior for the heterogeneity component. The intrinsic conditional autoregressive model (CAR) [23], in particular the Gaussian CAR prior, was used as the prior for the clustering component: each relative risk is independent of all the others conditional on a small set of "neighbours" that are influential in predicting the relative risk. Several definitions of neighbours can be used, the most common one is adjacency, that is all IRIS sharing a border with the IRIS of interest are defined as neighbours.

**Figure 4:**
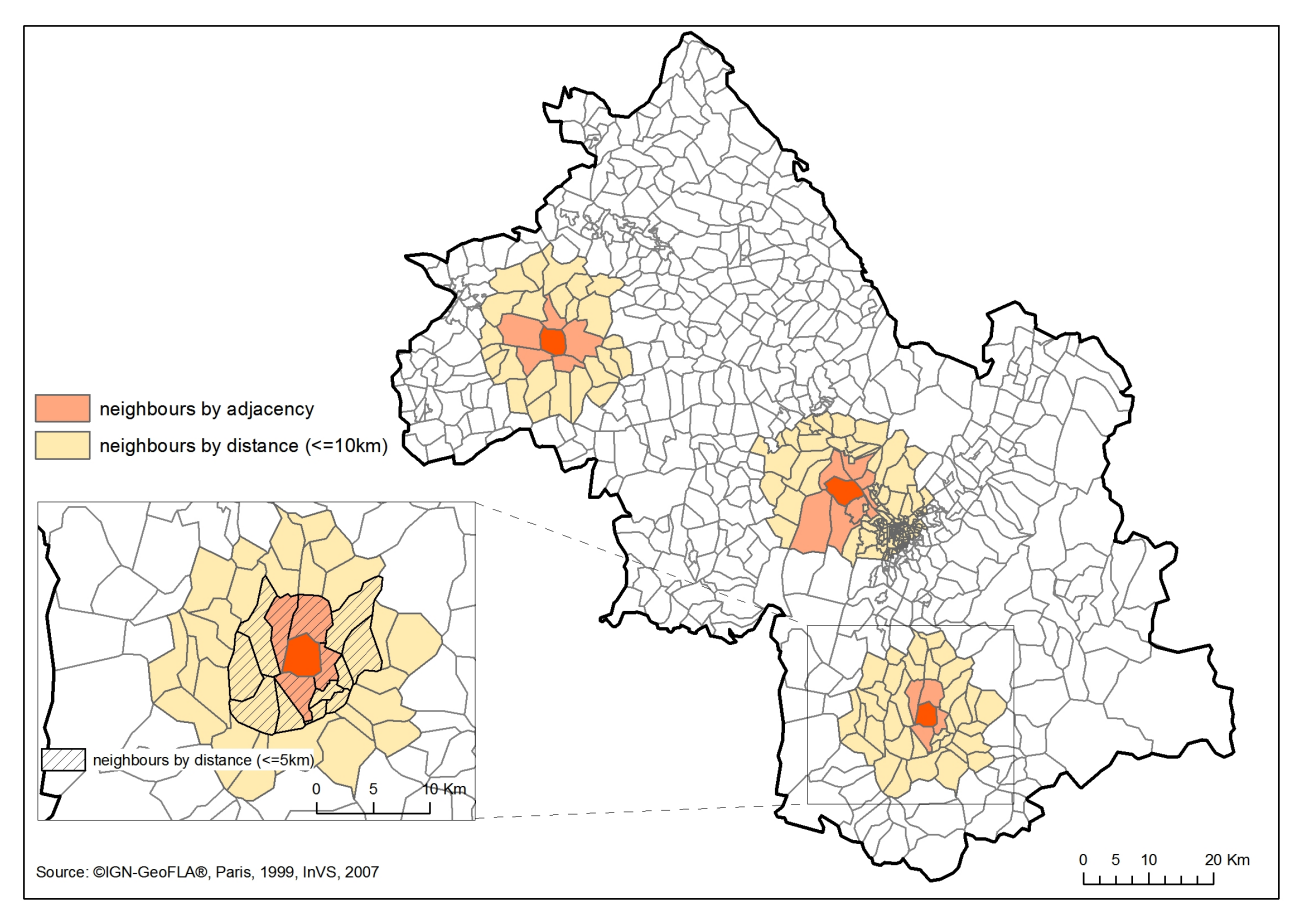
**Examples of the neighbourhoods defined by adjacency and distance for 3 IRIS- Isère department**.

**Figure 5:**
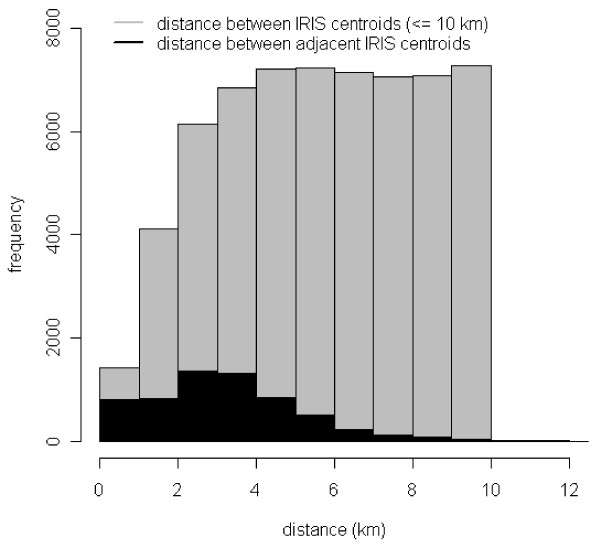
**Histogram of between IRIS centroid distances- 4 departments**. The number of neighbours is higher with the distance than the adjacency criterion.

For comparison we considered also an alternative set of neighbours defined by distance: two IRIS are neighbours if their centroids are less than 5 or 10 km apart. Figure 4 highlights for a few IRIS these different neighbourhoods. Figure 5 shows the histogram of the between IRIS centroid distances. Note that the sizes and shapes of the IRIS vary greatly. These sensitivity analyses allowed for spatial correlation at wider range than that implied by order 1 adjacency. The results obtained with the median-based CAR prior are also presented: the double-exponential (Laplacian) distribution is used rather than the Gaussian and the conditional distribution has its mode at the median of neighbouring values rather than at the mean [23,26]. This may be more appropriate when discontinuities between disease rates between areas are expected. As there is a potential interplay between the explained and unexplained part of the regression model, it is important to carry out such sensitivity analyses to different aspects of the modelling of the residual variations.

We have taken Gamma prior distributions for the precision parameters (reciprocal of the variance) of the heterogeneity and clustering terms. For both we have taken the non-informative *Γ*(0.5, 0.0005) [27]. The *Γ *(*a*, *b*) denotes the Gamma distribution with expectation equal to *a/b*. When only the heterogeneity term was in the model the prior distribution for its precision parameter was *Γ *(0.01, 0.01). For comparison we considered also the *Γ *(0.5, 0.0005) prior. Non-informative priors were taken for the other parameters, that is the intercept and the regression coefficients.

Posterior distributions did not have a closed analytical form. Therefore, simulation techniques based on Markov Chain Monte Carlo were used to obtain samples from the posterior distributions for the parameters. For each model a 200,000 iteration chain was run and posterior summaries were based on the final 7,000 iterations. Three chains with different initial values were run for each model and convergence was checked graphically [28].

Thus the analysis of the association between the risk of cancer and the exposure to MSWI was performed in two steps. First we fitted a Poisson regression, in particular a GAM, with inclusion of environmental covariates. Secondly, if necessary, we fitted a hierarchical Bayesian model in which extra-Poisson variability was explicitly modelled in terms of spatial and non-spatial components. The non-linear relationships estimated with the GAM models are here modelled by a piecewise regression with unknown knot positions- they were given a Gaussian prior with a large variance and the mean taken from the GAM models. This permitted modelling both overdispersion and non-linear relationships.

These analyses were carried out using the R package *mgcv *[29] and WinBUGS [28].

We note that the 4 departments included in this study are quite heterogeneous: Isère is a urban department, it is the most populated (around 850,000 inhabitants), the most exposed to MSWIs (50% of the exposed IRIS are in Isère) and with the highest values of exposure. On the contrary, Tarn is a rural department, it is the least populated (around 290,000 inhabitants), the least exposed (10% of the exposed IRIS are in Tarn) and with the lowest values of exposure. The covariables included in the models permit to account at least partially for this heterogeneity. A department's effect was also included in the models. The regression coefficients of the index of exposure by department were estimated (interaction of the department's effect and the index of exposure).

An additional sensitivity analysis that was carried out concerned the latency period. A 10-year latency period was considered for solid tumors taking as a reference 1995. This gave the exposure period ending in 1985. For comparison we considered a 15-year latency period, that is the exposure period 1972–1980. In this case 9 incinerators were included in the analysis.

## Results

### Index of exposure to MSWIs

**Figure 6:**
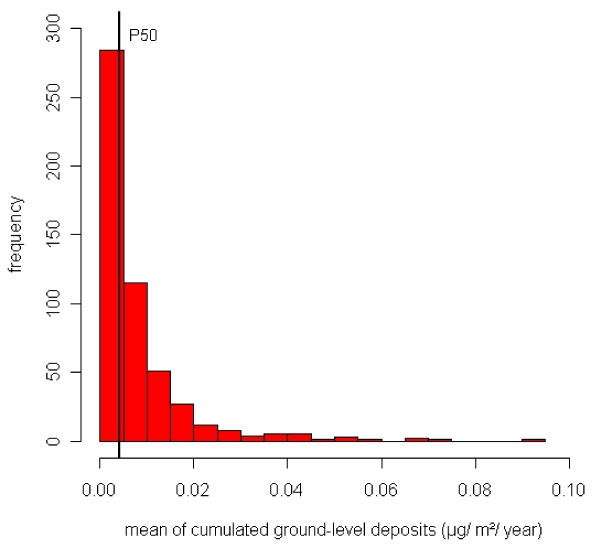
**Distribution of the average cumulative ground-level dioxin deposits within the modelisation grids**. It ranged from 2.04 × 10^-5 ^to 9.18 × 10^-2 ^*μ*g/m^2^/year. The median of this distribution, P50 = 4.25 × 10^-3^*μ*g/m^2^/year, is highlighted.

Figure 6 presents the distribution of the average cumulative ground-level deposits-distribution of the 520 IRIS that lie in the modelisation grids. As this distribution is highly right-skewed, a square root transformation was applied so that extreme values were not too influential in the fitting process. Thus the index of exposure to MSWIs is the square root of the mean of the cumulated ground-level deposits.

### Poisson regressions

**Figure 7:**
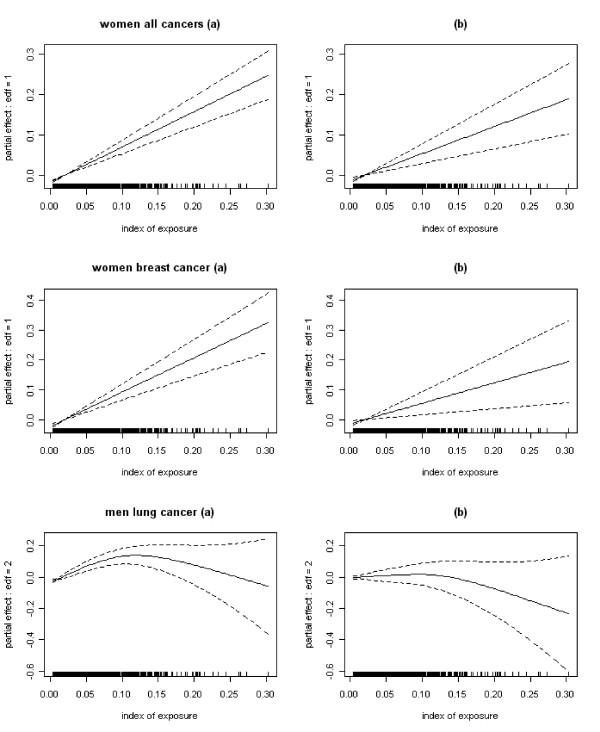
**Estimated associations (and their 95% CI) between the risk of cancer and the index of exposure to MSWIs (edf = degrees of freedom) for all cancers in women, breast cancer in women and lung cancer in men**. Associations estimated (a) without covariates in the model and (b) adjusting for the covariates. The highest exposed IRIS (index of exposure *> *0.16 = 95th percentile of the distribution of the exposed IRIS) are all exposed to the same incinerator, the incinerator of La Tronche in Isère. These IRIS have a small number of observed lung cancers in men compared to the expected number. The covariates included in the model do not fully explain this difference.

GAM models were fitted. For all the localisations of cancer studied at the exception of lung cancer in men, a nearly linear association between the risk of cancer and the index of exposure to incinerators was observed. Three examples of these associations are shown in Figure 7. The influence of the high values of the index of exposure on these associations was checked by removing some extreme values and little influence was found.

**Figure 8:**
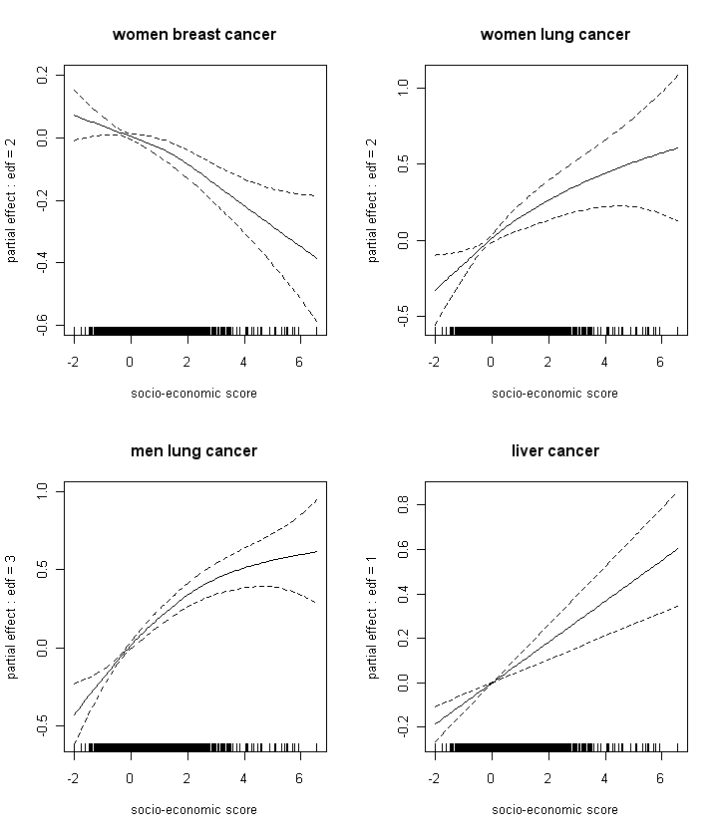
**Examples of the estimated associations (and their 95% CI) between the risk of cancer and the socio-economic score (edf = degrees of freedom)**.

Non-linear associations between the risk of cancer and the confounding variables were estimated for several localisations of cancer studied. Examples of the associations between the risk of cancer and the socio-economic score are shown in Figure 8. The higher the score the greater the deprivation, thus, for example, for women breast cancer the least deprived IRIS have a higher risk of cancer, while for liver cancer the most deprived have a higher risk of cancer.

The interest of using GAM models was highlighted in these figures: they allowed studying the forms of the associations, this could not be done by fixing a priori a linear association.

Table 1 presents the results of these Poisson regressions fitted with GAM taking into account the confounding variables for all cancers in women, all cancers in men, breast cancer in women and liver cancer. The results obtained with no covariates in the models are also presented. The department's effect is always included in the models. We note that when including the covariates in the model the coefficient for liver cancer more than doubles. The association between the risk of liver cancer and the index of exposure to MSWIs is positive and close to significance when the environmental covariates are in the model (p = 0.08) while it is positive but not significant when no covariates are in the model (p = 0.30). This is due to the fact that the rural/urban indicator is a strong confounding variable for the analysis of liver cancer and exposure to MSWIs. The risk of liver cancer is higher in rural IRIS than in urban ones while the exposure to incinerators is higher in urban IRIS than in rural ones. We believe that this variable can be seen as a proxy for alcohol consumption.

**Table 1:** Poisson regressions.

	Model 1 coefficient (95% CI) deviance; n – p	Model 2 coefficient (95% CI) deviance; n – p
All cancers in women	**0.879 (0.671, 1.087)** 3029; 2265	**0.671 (0.370, 0.971)** 2941; 2258
All cancers in men	**0.534 (0.316, 0.752)** 3782; 2265	0.214 (-0.092, 0.520) 3367; 2256
Breast cancer in women	**1.159 (0.826, 1.492)** 2596; 2265	**0.687 (0.231, 1.144)** 2522; 2260
Liver cancer	0.440 (-0.438, 1.318) 2348; 2265	1.119 (-0.131, 2.369) 2287; 2257

The estimates of regression coefficients and their 95% confidence intervals for the index of exposure to MSWIs, deviance and degrees of freedom.

The deviance divided by its degrees of freedom, n – p (number of observations – number of parameters), gives an estimate of the dispersion parameter: if it is > 1 it may indicate overdispersion. Coefficients statistically significant at the 5 per cent level are in bold.

Model 1: without covariates; Model 2: adjusting for the covariates

**Figure 9:**
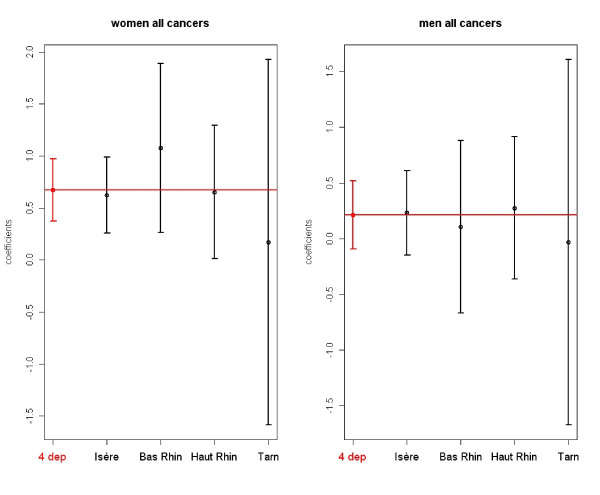
**Examples of the estimated regression coefficients and their 95% CI for the index of exposure to MSWIs: in red the coefficients presented in Table 1- Model 2- and in black the coefficients estimated by department**.

Figure 9 presents the regression coefficients for the index of exposure to incinerators and their 95% confidence intervals for all cancers in women and all cancers in men. The figure also presents the coefficients estimated by department. We note the weight of Isère. There is no significant difference between the coefficients estimated by department for Bas Rhin, Haut Rhin and Tarn and the coefficient estimated for Isère.

The sensitivity analysis that was carried out on the latency period considered a 15-year instead of a 10-year latency period. The positive associations we observed are maintained with this different latency period (results not shown).

As it can be seen from Table 1, although the covariates are taken into account, there is still evidence of overdispersion for the localisations of cancers studied.

### Hierarchical model

A hierarchical Bayesian model was interesting for all cancers in women, all cancers in men, breast cancer in women, lung cancer in men and liver cancer. And more specifically, a hierarchical Bayesian model with both heterogeneity and clustering terms was estimated for all cancers in women, breast cancer in women and all cancers in men, while a hierarchical Bayesian model with only the heterogeneity component was estimated for lung cancer in men and liver cancer. This was based on the comparison the DIC of hierarchical models with both heterogeneity and clustering terms to those with only the heterogeneity term.

As we have seen, several non-linear relationships between the risk of cancer and the index of exposure or the confounding variables were estimated. We fitted a piecewise regression with one knot whose position was not fixed a priori to account for these associations in the Bayesian hierarchical models.

Table 2 presents the results for all cancers and breast cancer in women. We can observe that the association between all cancers in women and the index of exposure to MSWIs remains positive and significant when the heterogeneity and clustering terms are added in the model. In particular, when adding the spatial term in the model the coefficient of the index of exposure gets smaller- due to confounding between exposure and location- and its standard deviation increases. The association between the risk of breast cancer and the exposure to MSWI remains positive and significant when the heterogeneity and clustering terms are added in the model. The standard deviation increases when adding the spatial term in the model. Overall, as expected, the hierarchical model gives higher standard errors as it does not underestimate the residual variability. Table 2 also presents the *p*_*D *_terms: they indicate strong structural information in the prior.

**Table 2:** Poisson regressions and hierarchical models.

	Model 2 coefficient (95% CI) DIC	Model 3 coefficient (95% CI) DIC; *p*_*D*_	Model 4 coefficient (95% CI) DIC; *p*_*D*_
All cancers in women	**0.671 (0.371, 0.971)** 12950	**0.670 (0.334, 1.003)** 12813; 367	**0.502 (0.083, 0.957)** 12776; 298
Breast cancer in women	**0.687 (0.230, 1.144)** 9751	**0.716 (0.217, 1.206)** 9738; 145	**0.680 (0.040, 1.315)** 9707; 121

The estimates of regression coefficients for the index of exposure to MSWIs, that is posterior means for the hierarchical models, their 95% confidence/credible intervals and DIC and *p*_*D *_terms for models comparison

Model 2: Poisson regression; Model 3: hierarchical model with heterogeneity component; Model 4: hierarchical model with both heterogeneity and spatial components.

For comparison different prior specifications of the clustering component were considered. Table 3 presents these results for all cancers and breast cancer in women. For all cancers in women, the prior model for the clustering component that is best supported by the data, that is with the smallest DIC, is the one with the neighbourhood defined by the 10 km distance. For breast cancer in women, the prior model for the clustering component that is best supported by the data is the one with the neighbourhood defined by the adjacency criterion. For both, all cancers and breast cancer in women, the prior that leads to most borrowing of strength across units, that is with the smallest *p*_*D*_, is the one with the neighbourhood defined by the 5 km distance. We note that the same results are obtained whether using the median based prior or the gaussian prior. Overall the results seem robust to the clustering prior specification.

**Table 3:** Bayesian hierarchical models with different priors for the clustering component.

	coefficient	(95% CI)	DIC; *p*_*D*_
All cancers in women			
*Gaussian CAR prior*			
adjacency	0.502	(0.083, 0.957)	12776; 298
distance ≤ 5 km	0.560	(0.185, 0.924)	12770; 277
distance ≤ 10 km	0.581	(0.235, 0.931)	12739; 289
*Median based CAR prior*			
adjacency	0.526	(0.071, 0.981)	12773; 292

Breast cancer in women			
*Gaussian CAR prior*			
adjacency	0.680	(0.040, 1.315)	9707; 121
distance ≤ 5 km	0.707	(0.076, 1.239)	9725; 94
distance ≤ 10 km	0.717	(0.176, 1.258)	9717; 128
*Median based CAR prior*			
adjacency	0.660	(0.052, 1.284)	9708; 116

The estimates of regression coefficients, their 95% credible intervals for the index of exposure to MSWIs, DIC and *p*_*D *_terms

## Concluding remarks

In studies of weak associations it is important to use advanced methods to better assess dose-response relationships with disease risk. In this work we used dispersion modelling, advanced GIS and statistical techniques to estimate the association between the risk of cancer and the exposure to incinerators.

As it is the case for most ecological studies on past exposures, no measurements were available of particles, metals and dioxins around the incinerators included in this study. The use of dispersion models associated with expert judgement to assess stack emissions represented the best surrogate for past exposure to incinerators. Expert judgement is an approach for soliciting informed opinions and is appropriate to assess retrospectively past emissions [30]. However, attention must be paid to the choice of the group of experts for the subjective nature of their judgement. In most epidemiological studies the exposure area and exposure status of the geographical units are usually defined by distance to the source. Dispersion models are still rarely used in studies of risk of disease and proximity to a point source of pollution [5,31,32]. In this work the use of a second generation Gaussian dispersion model accounting for complex field allowed a more reliable assessment of exposure status to incinerators than the calculation of the distance to the source. If measurements had been available it would have been interesting to combine the observed data and the estimates from dispersion models. This could be done using geostatistical methods of interpolation.

Geographical information systems are now an indispensable tool to carry out ecological studies. In this work it allowed defining the exposure to MSWIs and to road traffic at the IRIS level.

The non-linear relationships between the risk of cancer and the index of exposure to MSWIs or the covariables were taken into account fitting a GAM model. The distribution of the index of exposure was highly right-skewed. The influence of the high values was checked.

As in many geographical studies of rare disease incidence, there was clear evidence of extra-Poisson variability. It was the case for all cancers in women, all cancers in men, breast cancer in women, lung cancer in men and liver cancer. After fitting standard Poisson regressions, we have explicitly modelled the overdispersion in a hierarchical Bayesian framework. Bayesian hierarchical models with a heterogeneity and a clustering term allowed for unmeasured or unknown risk factors. In particular, modelling the clustering variation allowed accounting indirectly for those unmeasured risk factors that vary smoothly with location. That is it provided a flexible way of including the effect of location. And as we have seen, this was particularly interesting for the analysis of all cancers in women. The sensitivity of the results to the specification of the prior distributions was investigated. The results seemed robust. In the Bayesian hierarchical models, the non-linear relationships highlighted with the GAM were modelled by a piecewise regression with unknown knot positions. This allowed modelling both overdispersion and non-linear relationships.

A possible extension of the model would allow for exposure measurement error [33], that is taking into account that the modelled exposure is a surrogate for exposure and using expert opinion and epidemiological knowledge to model the uncertainty.

In this work the geographical unit is the IRIS: in urban zones it is a small geographical unit and we can suppose that there is little within-area variability of the risk factors, however in rural zones the IRIS coincides with the *commune *that can be quite extended. Thus, if possible, we would recommend obtaining and using information about the within-area distribution or variability of the risk factors [34].

This is an ecological study and the main limitations of this type of epidemiological analysis remain. In particular, no data was available on residential history leading to potential exposure misclassification. We also lacked information pertaining to individual potential confounders, such as occupational history, tobacco or alcohol consumption, diet, etc., that could confound the relationship between the risk of cancer and MSWI exposure. Thus, we cannot exclude the possibility of residual confounding.

## Competing interests

The authors declare that they have no competing interests.

## Authors' contributions

SG performed the statistical analysis and prepared the manuscript. CD carried out the atmospheric diffusion modelling and prepared the manuscript. PCC carried out the GIS part of the research. PEB supervised the study. PF organized data collection procedures, and managed the team. MC contributed with academic discussions and revised the manuscript. CD contributed with academic discussions and revised the manuscript. JFV contributed with academic discussions and revised the manuscript. SR contributed with academic discussions and revised the manuscript. All authors read and approved the final manuscript.
